# Supplementary material for: Role of NADPH Oxidases in Stroke Recovery
Source: Antioxidants (Basel). 2024 Aug 30;13(9):1065. doi: 10.3390/antiox13091065 (PMC11428334; doi:10.3390/antiox13091065)
Supplement: Supplementary file 1 [file antioxidants-13-01065-s001.zip › antioxidants-3142924-supplementary.pdf]

Supplemental Table S1. Results of literature searches in PubMed

To search for animal experimental studies, a comprehensive search of MEDLINE and PubMed databases was performed without restrictions on publication date or language, following the recommendations of the Systematic Review Center for Experimental Animal Research (SYRCLE). All articles were solicited from the databases using the searching terms “rat or mouse”, “stroke recovery”, “stroke repair” and “NADPH oxidase”. A key-word search for “rat/mouse/stroke/recovery/repair/NADPH oxidase” were used med-ical subject headings (MeSH) terms and all fields’ terms. Of the 21 articles identified in MEDLINE and PubMed (as of May 2024), 7 articles that did not meet the study criteria were excluded (two in vitro studies and five articles on other diseases). Finally, 14 articles were included in this review. The publication dates of these articles ranged from December 1, 2004 to May 14, 2024.

Search number	Query	Search Details	Results
7	#5 or #6	(("rats"[MeSH Terms] OR "rats"[All Fields] OR "rat"[All Fields] OR ("mice"[MeSH Terms] OR "mice"[All Fields] OR "mouse"[All Fields] OR "mouse s"[All Fields] OR "mouses"[All Fields])) AND (("stroke"[MeSH Terms] OR "stroke"[All Fields] OR "strokes"[All Fields] OR "stroke s"[All Fields]) AND ("recoveries"[All Fields] OR "recovery"[All Fields])) AND ("nadph oxidases"[MeSH Terms] OR ("nadph"[All Fields] AND "oxidases"[All Fields]) OR "nadph oxidases"[All Fields] OR ("nadph"[All Fields] AND "oxidase"[All Fields]) OR "nadph oxidase"[All Fields])) OR (("rats"[MeSH Terms] OR "rats"[All Fields] OR "rat"[All Fields] OR ("mice"[MeSH Terms] OR "mice"[All Fields] OR "mouse"[All Fields] OR "mouse s"[All Fields] OR "mouses"[All Fields])) AND (("stroke"[MeSH Terms] OR "stroke"[All Fields] OR "strokes"[All Fields] OR "stroke s"[All Fields]) AND ("repairability"[All Fields] OR "repairable"[All Fields] OR "repaire"[All Fields] OR "repaired"[All Fields] OR "repairment"[All Fields] OR "wound healing"[MeSH Terms] OR ("wound"[All Fields] AND "healing"[All Fields]) OR "wound healing"[All Fields] OR "repair"[All Fields] OR "repairing"[All Fields] OR "repairs"[All Fields])) AND ("nadph oxidases"[MeSH Terms] OR ("nadph"[All Fields] AND "oxidases"[All Fields]) OR "nadph oxidases"[All Fields] OR ("nadph"[All Fields] AND "oxidase"[All Fields]) OR "nadph oxidase"[All Fields]))	21
6	#1 and #3 and #4	("rats"[MeSH Terms] OR "rats"[All Fields] OR "rat"[All Fields] OR ("mice"[MeSH Terms] OR "mice"[All Fields] OR "mouse"[All Fields] OR "mouse s"[All Fields] OR "mouses"[All Fields])) AND (("stroke"[MeSH Terms] OR "stroke"[All Fields] OR "strokes"[All Fields] OR "stroke s"[All Fields]) AND ("repairability"[All Fields] OR "repairable"[All Fields] OR "repaire"[All Fields] OR "repaired"[All Fields] OR "repairment"[All Fields] OR "wound healing"[MeSH Terms] OR ("wound"[All Fields] AND "healing"[All Fields]) OR "wound healing"[All Fields] OR "repair"[All Fields] OR "repairing"[All Fields] OR "repairs"[All Fields])) AND ("nadph oxidases"[MeSH Terms] OR ("nadph"[All Fields] AND "oxidases"[All Fields]) OR "nadph oxidases"[All Fields] OR ("nadph"[All Fields] AND "oxidase"[All Fields]) OR "nadph oxidase"[All Fields])	10

5	#1 and #2 and #4	("rats"[MeSH Terms] OR "rats"[All Fields] OR "rat"[All Fields] OR ("mice"[MeSH Terms] OR "mice"[All Fields] OR "mouse"[All Fields] OR "mouse s"[All Fields] OR "mouses"[All Fields])) AND (("stroke"[MeSH Terms] OR "stroke"[All Fields] OR "strokes"[All Fields] OR "stroke s"[All Fields]) AND ("recoveries"[All Fields] OR "recovery"[All Fields])) AND ("nadph oxidases"[MeSH Terms] OR ("nadph"[All Fields] AND "oxidases"[All Fields]) OR "nadph oxidases"[All Fields] OR ("nadph"[All Fields] AND "oxidase"[All Fields]) OR "nadph oxidase"[All Fields])	15
4	NADPH oxidase	"nadph oxidases"[MeSH Terms] OR ("nadph"[All Fields] AND "oxidases"[All Fields]) OR "nadph oxidases"[All Fields] OR ("nadph"[All Fields] AND "oxidase"[All Fields]) OR "nadph oxidase"[All Fields]	27,903
3	stroke repair	("stroke"[MeSH Terms] OR "stroke"[All Fields] OR "strokes"[All Fields] OR "stroke s"[All Fields]) AND ("repairability"[All Fields] OR "repairable"[All Fields] OR "repaire"[All Fields] OR "repaired"[All Fields] OR "repairment"[All Fields] OR "wound healing"[MeSH Terms] OR ("wound"[All Fields] AND "healing"[All Fields]) OR "wound healing"[All Fields] OR "repair"[All Fields] OR "repairing"[All Fields] OR "repairs"[All Fields])	11,681
2	stroke recovery	("stroke"[MeSH Terms] OR "stroke"[All Fields] OR "strokes"[All Fields] OR "stroke s"[All Fields]) AND ("recoveries"[All Fields] OR "recovery"[All Fields])	34,588
1	rat or mouse	"rats"[MeSH Terms] OR "rats"[All Fields] OR "rat"[All Fields] OR "mice"[MeSH Terms] OR "mice"[All Fields] OR "mouse"[All Fields] OR "mouse s"[All Fields] OR "mouses"[All Fields]	3,780,173
